# Hypoglycemic, hypolipidemic and hepato-protective effect of bee bread in streptozotocin-induced diabetic rats

**DOI:** 10.22038/AJP.2021.17748

**Published:** 2021

**Authors:** Meryem Bakour, Nawal El Menyiy, Asmae El Ghouizi, Badiaa Lyoussi

**Affiliations:** 1 *Laboratory of Natural Substances, Pharmacology, Environment, Modeling, Health and Quality of life (SNAMOPEQ), Sidi Mohamed Ben Abdellah University, Fez, Morocco*

**Keywords:** Diabetes, Bee bread, Hyperlipidemia, Liver

## Abstract

**Objective:**

This study aims to shed a new light on pharmacological effects of bee bread as a product of the hive through examination of the effect of itsethyl acetate extract onhyperglycemia, dyslipidemia, and liver dysfunction induced by streptozotocin.

**Materials and Methods:**

The bee bread ethyl acetate extract was analyzed for total phenolics, flavonoids, and the antioxidant activities using total antioxidant capacity, 2, 2- diphenyl-1-picrylhydrazyl (DPPH), 2, 2'-azino-bis (3-ethylbenzothiazoline-6-sulfonic acid (ABTS), and reducing power assays. *In vivo* study was carried out on thirty-six rats divided into control or diabetic rats, received daily for 15 days distilled water (10 ml/kg), or ethyl acetate extract of bee bread (100 mg/kg), or glibenclamide (2.5 mg/kg). The protective effect of bee bread against metabolic changes induced by streptozotocin in Wistar rats, was evaluated by checking the blood glucose levels, lipid profile, atherogenic index, coronary risk index, cardiovascular risk index, body weight and hepatic enzyme markers in normal and diabetic rats. Glibenclamide was used as standard drug to compare the efficacy of bee bread.

**Results:**

The results indicate that bee bread ethyl acetate extract has a high content of phenolics and flavonoids and a strong antioxidant activity. Glycemia, lipid profile and hepatic enzymes were modified in diabetic rats. These modifications were ameliorated after the treatment withbee bread extract which was more potent than glibenclamide.

**Conclusion:**

In summary, ethyl acetate extract of bee bread possesses effective glycemia lowering effects and representsa natural source of new bioactive molecules for future therapy of hyperglycemia, hyperlipidemia and liver dysfunction.

## Introduction

The nutrients necessary for the survival and health maintenance of bee colony come from two main sources namely, nectar and pollen. 

Nevertheless, bees do not consume these foods directly; but they induce biochemical changes, the nectar turns into honey andthepollen turns into bee bread (Dolezal and Toth, 2018 ▶; Vaudo et al., 2015 ▶).

The process of forming the bee bread begins with harvesting the pollen of flowers, which is mixed by bee’s with saliva that contains its own digestive enzymes and nectar or honey.The pollen from flowers is transformed into a pollen ball or bee pollen, it is stored in the rear legs of the bee where there is a pollen basket and then carried to the hive. At this stage, other bees continue the mission by filling the ¾ of the alveoli of the hive by the balls of the pollen and the ¼ which remains by the honey. Finally, a thin layer of wax is added to cover the cells and to protect the pollen from the oxygen. The bee bread isformed after an anaerobic lactic fermentation process, which makes it richer in nutrients and more digestible than bee pollen (Barene et al., 2014 ▶; Kieliszek et al., 2018 ▶) .

Diabetes is a chronic epidemic disease affecting millions of people worldwide;it is basically classified into two major types: type 1 diabetes or insulin-dependent diabetes and type 2 diabetes or non-insulin-dependent diabetes. Type 1 diabetes is due to autoimmune destruction of pancreatic beta cells, leading to insulin deficiency, and it usually begins to manifest in childhood and early adulthood (Chatterjee et al., 2017 ▶; DiMeglio et al., 2018 ▶).

Streptozotocin is the most preferred chemical used for modelling human diabetes in laboratory animals. It is absorbed by pancreatic β cells via the GLUT_2_ glucose transporter. The main reason for STZ-induced β-cell death is the alkylation of DNA that causes the formation of poly-ADP-ribosylationand a depletion of ATP and NAD^+ ^(Szkudelski, 2001 ▶).

The main objective of this study was to investigate the hypoglycemic and hypolipidemic effect of bee bread in diabetes. To our knowledge, this study is the first on the anti-diabetic effect of this hive product.

## Materials and Methods

**Bee breadsample and extraction**

Bee bread used in this study was produced in Imouzermarmoucha, Morocco. The bee bread extract was prepared by liquid-liquid extraction usingwater and ethyl acetate;the ethyl acetate extract was placedin a rotary evaporator then, the desired concentration (100 mg/kg) was obtained after adding distilled water.

The bee bread used in this study was classified as multifloral. Phenolic compounds profile, minerals, tocopherols, fatty acids, and sugar composition of the bee bread sample was reported in our previous study (Bakour et al., 2019 ▶).

**Quantification of total phenolic contents**

The total phenolic contents was determined spectrophotometrically usingMărghitaş et al. method (Mărghitaş et al., 2009 ▶). The phenolic content of bee bread was determined using a calibration curve. The results areexpressed as mg of gallicacid equivalent per gram of bee bread ethyl acetate extract.

**Total flavonoid content**

Total flavonoid content was determined spectrophotometrically usingKong et al.method (Kong et al., 2012 ▶). The absorbance was read at 510 nm using a calibration curve plotted forquercetin. The results areexpressed as mg of quercetin equivalent/g of bee bread ethyl acetate extract. 

**Total antioxidant capacity**

The total antioxidant capacity of bee bread extract was evaluated spectrophotometrically according to the method of Zengin et al. (Zengin et al., 2013 ▶). The antioxidant capacity of ethyl acetate extract of bee bread was evaluated as equivalent of ascorbic acid (mg Eq AA/g of bee bread ethyl acetate extract). 

**Free radical scavenging activity (DPPH)**

Free radical scavenging activity was done according to Miguel et al. method (Miguel et al., 2014). BHT (butylated hydroxytoluene) was used as positive control, and the absorbance was taken at 517 nm after one hour. Tests were carried out in triplicate and the scavenging activity was estimated based on the percentage of DPPH radical scavenged using the following equation IC_50_=[(A_0_-A_1_/A_0_)×100], where A_0_ is the absorbance of a negative control (blank sample containing the same amount of solvent and DPPH solution) and A_1_ is the absorbance of the sample.

**2, 2’-azino-bis (3-ethylbenzothiazoline-6-sulphonic acid (ABTS**) 

ABTS radical scavenging activity was evaluated spectrophotometrically using Silva et al. method (Silva et al., 2013 ▶). The absorbance at 734 nm was read after 6 min. All tests were carried out in triplicate. The capability to scavenge the ABTS radical was calculated using the following equation: IC_50_=[(A_0_-A_1_/A_0_)×100], where A_0_ is the absorbance of a negative control (blank sample containing the same amount of solvent and ABTS solution) and A_1_ is the absorbance of the sample. 

**Reducing Power (RP)**

The reducing power was determined spectrophotometrically using Padmanabhan and Jangle method (2012) ▶. The absorbance was measured at 700 nm; ascorbic acid was used as a positive control. The results are represented as the extract concentration providing 0.5 of absorbance (EC_50_), calculated from the graph obtained by plotting the absorbance at 700 nm, against the corresponding extract concentration (Padmanabhan and Jangle, 2012 ▶).

**Animals**

Thirty-six male Wistar rats (167.04±28.95 g) were chosen at random for the experiment. Internationally accepted principles for the use and care of laboratory animals have been followed for the care and handling of rats as per the guidelines of the European Community (EEC Directive of 1986; 86/609/EEC). The approval from the Ethical Committee, Faculty of Sciences, Fez, Morocco was obtained (USMBA-SNAMOPEQ 2017–03).

**Induction of experimental diabetes**

Diabetes was artificially induced by intravenous injection of streptozotocin (STZ) (Sigma, St. Louis, MO) (60 mg/kg dissolved in citrate buffer, 0.1 M, pH 4.5) through the tail vein (vena caudalis major). After 3 days, blood glucose of rats injected with STZ was measured spectrophotometrically using the glucose oxidase method to confirm hyperglycemia. The rats selected for the study were those with fasting blood glucose levels greater than 250 mg/dl (El Hilaly and Lyoussi, 2002 ▶).

**Experimental design**

Thirty-six rats were chosen at random and divided into six groups (n=6 per group). Group 1: non-diabetic rats that received distilled water (10 ml/kg); Group 2: non-diabetic rats that received ethyl acetate extract of bee bread (BBE) at a dose of 100 mg/kg; Group 3: non-diabetic rats that received glibenclamide (GLB) (BENCLAMID® PROPHARMA) at a dose of 2.5 mg/kg, the dose of GLB was chosen following the protocol described by (Chukwunonso Obi et al., 2015 ▶); Group 4: diabetic rats that received distilled water (10 ml/1 kg); Group 5: diabetic rats that were treated with BBE at a dose of 10 0mg/kg; and Group 6: diabetic rats that were treated with GLB 2.5 mg/kg.

BBE, water and GLB were administered orally once daily to each rat for a total of 15 days. Blood glucose was monitored for 3 hr was done. Blood samples were taken on the last day of the experiment from retro-orbital puncture of all Wistar rats under anesthesia (by inhaling a cotton ball soaked in diethyl ether) using capillary tubes then centrifuged, and the plasma was conserved until analysis.

**Biochemical analysis**

Plasma levels of glucose, triglycerides (TGs), cholesterol (TC), low density lipoprotein (LDL-C), very low density lipoprotein (VLDL), and high density lipoprotein (HDL-C), and the activities of alanine aminotransferase (ALT), aspartate aminotransferase (AST); Lactate dehydrogenase (LDH) and alkaline phosphatase (ALP) of all groups were analyzed. Atherogenic Index (AI), Coronary Risk Index (CRI) and Cardiovascular Risk Index (CVRI) were calculated using the following formulae: AI=LDL cholesterol/HDL cholesterol; CRI=TC/HDL cholesterol; CVRI=TGs/HDL cholesterol (Erejuwa et al., 2016 ▶)

**Statistical Analysis**

The results are presented as mean±SD. Statistical analysis was carried out by GraphPad Software 5, using One-way analysis of variance followed by *Post hoc* Tukey’s multiple comparison test. Student t test was used to compare the change in body weight between day 1 and day 15.A p<0.05 was considered significant.

## Results

**Antioxidant content and antioxidant activities of bee bread**

The phenolic content as shown in Table 1 was 27.27±0.38 mg Eq GA/g, flavonoids was 5.29±0.27 mg Eq quercetin/g and total antioxidant capacity was 65.44±6.34 mg eq AA/g. The IC_50_/EC_50_ of antioxidant activity of BBE was 1.52±0.021 mg/ml by ABTS, 0.43±0.02 mg/ml by DPPH, and 0.71±0.05 mg/ml by reducing power.

**Effect of daily oral administration of BBE and GLB on glycemia in the diabetic and non-diabetic rats**

The results presented in Table 2 show that STZ caused a very significant rise in blood glucose levels. On the other hand, the results of the diabetic groups of rats treated with BBE showed a decrease in blood glucose reaching the normal value from the third day of treatment, while the results obtained with the reference drug GLB showed a decrease in blood glucose, but it remained significantly higher than the non-diabetic group that received distilled water.

**Glycemia changes during 3 hr after a single oral administration of BBE and GLB in the diabetic and non-diabetic rats**

Table 3 presents blood glucose levels during 3 hr. As shown in the results, after one hour and a half, the blood glucose was lowered better in the group treated with BBE than that of the group treated with GLB, and after 3 hr the decrease of glycemia continued to reach 200±41.36 mg/dl with BBE and (226±28.28 mg/dl) with GLB.

**Table 1:** The phenolic and flavonoids content and the antioxidant activities of bee bread *in vitro* (TAC, DPPH, ABTS and RP).

	**Phenolics** **mg EqGA/g**	**Flavonoids** **mg EqQ/g**	**TAC** **mg EqAA/g**	**ABTS** **mg/ml**	**DPPH** **mg/ml**	**RP** **mg/ml**
**BBE**	27.27±0.38	5.29±0.27	65.44±6.34	1.52±0.021	0.43±0.02	0.71±0.05
***BHT***	-	-	-	0.002±0.001	0.01±0.002	NT
***Ascorbic acid***	-	-	-	NT	NT	0.004±0.001

Data are expressed as mean±SD. NT=not tested; TAC=total antioxidant capacity; ABTS=Azino-bis (3-ethylbenzothiazoline-6-sulphonic acid; DPPH=2, 2-diphenyl-1-picrylhydrazyl; RP=reducing power; mg EqGA/g=mg equivalent Gallicacid/g; mgEqQ/g=mg equivalent Quercetin/g; mg EqAA/g=mg equivalent ascorbicacid/g.

**Table 2:** Effect of daily oral treatment with ethyl acetate bee bread extract and glibenclamide on blood glucose level in normal and STZ-diabetic rats

Type of group	Interventions	Blood glucose levels (mg/dl)-Time (days)					
		Day 1	Day 3	Day 6	Day9	Day 12	Day 15
Non-diabetic rats	DW	108±5***^b^	110±4***^b^	107±6***^b^	99±8***^b^	98±7***^b^	110±5.77***^b^
	BBE	113±3***^b^	112±5***^b^	112±2***^b^	111±4***^b^	110±2***^b^	98±2***^b^
	GLB	105±4***^b^	104±3***^b^	102±2***^b^	100±2***^b^	97±3***^b^	98±1***^b^
Diabetic rats	STZ+DW	386±23***^a^	400±4***^a^	398±5***^a^	394±7***^a^	402±3***^a^	409±60***^a^
	STZ+BBE	410±45***^a^***^c^	120±22***^a^	111±30***^b^	118±10*^a^***^b^	108±12***^b^	10±18***^b^
	STZ+GLB	343±13***^a^	220±25***^a^***^c^	230±31***^a^***^b^***^c^	179±19***^a^***^b^***^c^	160±36***^a^ ***^b^***^c^	181±11***^a^***^b^***^c^

Data are expressed as mean±SD; n=6 per group; ***p<0.001; DW=distilled water; BBE=bee bread extract; GLB=glibenclamide; STZ+DW=Streptozotocin diabetic group received distilled water; STZ+BBE=Streptozotocin diabetic group received BBE; STZ+GLB=Streptozotocin diabetic group received GLB; (a): comparison of all groups with the group received distilled water (DW); (b): comparison of all groups with STZ-diabetic group received distilled water (STZ+DW); (c): comparison between STZ-diabetic group received BBE (STZ+BBE) and STZ-diabetic group received GLB (STZ+GLB).

**Table 3:** Effect of single oral administration of ethyl acetate bee bread extract and glibenclamide on blood glucose level for 3 hr in normal and STZ-diabetic rats

Type of group	Interventions	Blood glucose levels (mg/dl)-Time (Hours)		
		0hr	1.5 hr	3hr
Non-diabetic rats	DW	95.00±9***^b^	103.33±15***^b^	84.33±10***^b^
	BBE	92.25±14***^b^	92.00±12***^b^	91.50±10***^b^
	GLB	93.50±8***^b^	95.50±12***^b^	109.00±11***^b^
Diabetic rats	STZ+DW	530±16.76***^a^	540±46.46***^a^	573±14.57***^a^
	STZ+BBE	525±51.61***^a^	522±28.99***^a^***^b^	200±41.36***^a^***^b^
	STZ+GLB	550±70.71***^a^	475±31.81***^a^*^b^	226±28.28***^a^***^b^

Data are expressed as mean±SD; n=6 per group; *p<0.05; ***p<0.001; DW=distilled water; BBE=bee bread extract; GLB=glibenclamide; STZ+DW=Streptozotocin diabetic group received distilled water; STZ+BBE=STZ-diabetic group received BBE; STZ+GLB=STZ-diabetic group received GLB; (a): comparison of all groups with the group received distilled water (DW); (b): comparison of all groups with STZ-diabetic group received distilled water (STZ+DW); (c): comparison between STZ-diabetic group received BBE (STZ+BBE) and STZ-diabetic group received GLB (STZ+GLB).

**Effect of daily oral administration of BBE and GLB on body weight in the diabetic and non-diabetic rats**

The results summarized in Table 4 show that after 15 days of STZ injection, the body weight was significantly reduced in the group of diabetic rats that received distilled water and in the group of diabetic rats treated with GLB, while no significant decreases were recorded in the group of diabetic rats treated with BBE.

**Effect of daily oral administration of BBE and GLB on lipid profile in the diabetic and non-diabetic rats**

The levels of TC, LDL-C, TG, VLDL and HDL-C, in plasma of non-diabetic and diabetic rats are shown in Table 5. Diabetic rats showed a significant increase in TC, TG, LDL-C, and VLDL, and a significant decrease in plasma HDL-C level. While treatment with GLB and BBE kept the values of TC, LDL-C, TG, VLDL and HDL-C, near normal values.

**Table 4:** Effect of daily oral treatment of ethyl acetate bee bread extract and glibenclamide on body weight in normal and STZ-diabetic rats

**Type of group**	**Interventions**	**Body weight (g)**	
		Day 0 (baseline)	Day 15
**Non diabetic rats**	DW	153.33±11.37	160±10
	BBE	145±95	169.5±32
	GLB	185.5±9.19	184±19.79
**Diabetic rats**	STZ+ DW	171±12.72	94.5±4.5**
	STZ+ BBE	197.75±32.62	152.75±41.11
	STZ+ GLB	146±8.45	116±5.65*

Data are expressed as mean±SD; n=6 per group; *p<0.05; **p<0.01; DW=distilled water; BBE=bee bread extract; GLB=glibenclamide; STZ+DW=Streptozotocin diabetic group received distilled water; STZ+BBE=STZ-diabetic group received BBE; STZ+GLB=STZ-diabetic group received GLB; the comparison was made between day 0 and day 15 using t-test.

**Table 5:** Effect of repeated daily oral treatment with the ethyl acetate bee bread extract and glibenclamide on blood TC, TG, LDL-C, HDL-C, and VLDL-C levels and atherogenicindexin normal and STZ-diabetic rats

Type of group	Interventions	TC**(mg/dl)**	HDL-C**(mg/dl)**	TGs**(mg/dl)**	LDL-C **(mg/dl)**	VLDL**(mg/dl)**
Non-diabetic rats	DW	161±7***^b^	55±4.93***^b^	150.3±5.47***^b^	74.97±10***^b^	31.06±1.09***^b^
	BBE	160.66±8***^b^	54.66±2.9***^b^	146±3.34***^b^	76.8±9***^b^	29.09±2.46***^b^
	GLB	164.33±7.88***^b^	55.33±3.38**^b^	156.33±2.88***^b^	77.73±10.44***^b^	31.20±2.17***^b^
Diabetic rats	STZ+DW	246.33±3.17***^a^	26.66±2.18***^a^	226.33±8.98***^a^	174.4±4.93***^a^	45.26±3.79***^a^
	STZ+BBE	170±5***^b^	46.33±2.72*^a^***^b^	163±2.64***^b^	91.06±4.08*^a^***^b^	32.60±1.52***^b^
	STZ+GLB	166.33±10***^b^	42.66±7***^a^***^b^	165.66±3.84***^b^	90.53±3.80*^a^***^b^	33.13±1.76***^b^

Data are expressed as mean±SD; n=6 per group; *p<0.05; **p<0.01; ***p<0.001; DW=distilled water; BBE=bee bread extract; GLB=glibenclamide; STZ+DW=STZ-diabetic group received distilled water; STZ+BBE=STZ-diabetic group that received BBE; STZ+GLB=STZ-diabetic group that received GLB; (a): comparison of all groups with the group that received distilled water (DW); (b): comparison of all groups with STZ-diabetic group that received distilled water (STZ+DW); (c): comparison between STZ-diabetic group that received BBE(STZ+BBE) and STZ-diabetic group that received GLB (STZ+GLB)

**Effect of daily oral administration of BBE and GLB on the atherogenic index, cardiovascular risk index and cardiovascular risk index in the diabetic and non-diabetic rats**

Table 6 shows the results of Atherogenic Index (AI), Coronary Risk Index (CRI) and Cardiovascular Risk Index (CVRI);the elevation of these indexes was observed in the untreated diabetic rats, while for the treated diabetic rats, (AI), (CRI) and (CRVI) values obtained were near those obtained in the group of non-diabetic rats that received distilled water.

**Effect on hepatic enzymes**

Table 7 shows AST, ALT, LDH and ALP levels in plasma of normal and diabetic rats. The diabetic rats showed a significant increase in these liver enzymes levels. While with the use of GLB and BBE a significant lowering of these enzymes was observed. 

**Table 6:** Effect of repeated daily oral treatment with the ethyl acetate Bee bread extract and glibenclamide on the Coronary Risk Index (CRI), atherogenicindex (AI), and Cardiovascular Risk Index (CVRI) in normal and STZ-diabetic rats

Type of group	Groups	CRI** (units)**	AI**(units)**	CVRI**(units)**
Non-diabetic rats	DW	2.92±0.33***^b^	1.34±0.34***^b^	2.73±1.1***^b^
	BBE	2.92±0.22***^b^	1.41±0.27***^b^	2.67±1.15***^b^
	GLB	3.00±0.29***^b^	1.43±0.26***^b^	2.82±0.26***^b^
Diabetic rats	STZ+DW	9.34±0.67***^a^	6.60±0.44***^a^	8.48±4.11***^a^
	STZ+BBE	3.69±0.25***^b^*^a^	1.99±0.21***^b^**^a^	3.51±0.9***^b^
	STZ+GLB	3.90±0.12***^b^***^a^	2.12±0.11***^b^***^a^	3.88±0.54**^b^

Data are expressed as mean±SD; n=6 per group; *p<0.05; **p<0.01; ***p<0.001; DW=distilled water; BBE=bee bread extract; GLB=glibenclamide; STZ+DW=Streptozotocin diabetic groupthatreceived distilled water; STZ+BBE=STZ-diabetic group that received BBE; STZ+GLB=STZ-diabetic group that received GLB; (a): comparison of all groups with the group that received distilled water (DW); (b): comparison of all groups with STZ-diabetic group that received distilled water (STZ+DW).

**Table 7:** Effect of repeated daily oral treatment with the ethyl acetate Bee bread extract and glibenclamide on blood AST, ALT, LDH, and ALP levels in normal and STZ-diabetic rats

Type of group	Interventions	AST **(U/L)**	ALT **(U/L)**	LDH **(U/L)**	ALP **(U/L)**	
Non-diabetic rats	DW	130±5***^b^	58±6***^b^	580±20***^b^	120±11***^b^	
	BBE	125±3***^b^	59±7***^b^	577±4***^b^	116±14***^b^	
	GLB	128±4***^b^	57±5***^b^	579±7***^b^	119±12***^b^
Diabetic rats	STZ+DW	221±10***^a^	89±7***^a^	820±50***^a^	224±5***^a^	
	STZ+BBE	148±12***^b^	60±2***^b^	600±15***^b^	150±10***^b^	
	STZ+GLB	160±8**^a^***^b^	62±3***^b^	610±12***^b^	147±12***^b^	

Data are expressed as mean±SD; n=6 per group; **p<0.01; ***p<0.001; DW=distilled water; BBE=bee bread extract; GLB=glibenclamide; STZ+DW=STZ-diabetic groupthatreceived distilled water; STZ+BBE=STZ-diabetic group that received BBE; STZ+GLB=STZ-diabetic group that received GLB; (a): comparison of all groups with the group that received distilled water (DW); (b): comparison of all groups with STZ-diabetic group that received distilled water (STZ+DW).

## Discussion

The findings of the present study showed that the administration of a single dose of STZ causes a very significant elevation of glycemia (p<0.001); these results are in agreement with previous studies according to which a single dose of STZ caused damage in pancreas β-cells, leading to diabetes in rats (Dimo et al., 2007 ▶; El Hilaly and Lyoussi, 2002 ▶). The mechanism of action of GLB is well known, it is a secret agogue that stimulates insulin secretion from pancreatic β cells by sensitizing them to the action of glucose, it binds to a specific receptor in the

β cell (SUR1) and inhibits potassium efflux by closing ATP-dependent potassium channels (KATP); therefore, the results observed in this study for GLB are explained by the fact that STZ does not cause complete destruction of the β cells, and GLB stimulates insulin secretion in the residual β cells of these diabetic rats(de Wet and Proks, 2015 ▶). Concerning BBE, the blood glucose lowering effect was more potent than that observed for GLB. To explain this effect, several possible scenarios can be proposed and must be verified by future studies, such as an effect of increased glucose utilization via the insulin sensitization in peripheral tissues like that observed by taurine, (Nandhini et al., 2004 ▶) an insulin-like effect such as that observed by Singh et al. in diabetic rats treated with an Indian plant *Cinnamomumtamala* and *Aloe vera, *(Singh et al., 2015 ▶) an effect caused via decreased glucose uptake by the small intestine, (Li et al., 2005 ▶) or via the normalization of hepatic gluconeogenic enzymes (Pari and Venkateswaran, 2004 ▶).

There is evidence that increased TG and reduced HDL-C levels are the main features of dyslipidemia in diabetics. Hypertriglyceridemia in diabetes may result from increased VLDL production and impaired catabolism of TG -rich particles. Lipoprotein lipase, a key enzyme in the elimination and breakdown of TGs from the circulation, enzyme is alleviated by both insulin deprivation and insulin resistance. The abnormally high concentration of plasma lipids in diabetes is mainly due to the increased mobilization of free fatty acids from peripheral deposits, as insulin inhibits hormone-sensitive lipase (Garg, 1994 ▶).

HDL-C is a protector against atherosclerosis, it reverses the transport of cholesterol, inhibiting LDL-C oxidation and neutralizing the atherogenic effects of oxidized LDL-C. A large increase in LDL-C and VLDL-C can lead to a decrease in HDL-C, as there is a reciprocal relationship between the concentration of VLDL-C and HDL-C. The decrease in the HDL-C levels may also be due to a deficiency in lecithin cholesterol acyltransferase (Chandramohan et al., 2010 ▶). 

The liver is an organ that plays an important role in homeostasis of glucose and lipids; this homeostasis is severely affected by diabetes (Postic et al., 2004 ▶). The increase of LDH, ALP and hepatic transaminase levels is an index of liver cell injury. The results of this study revealed a significant elevation of these liver enzymes in diabetic rats. These results are identical to those obtained in other studies (Farokhi et al., 2012 ▶; Kazemian et al., 2015 ▶; Zarei et al., 2015 ▶). However, a significant decrease was observed in these hepatic enzymes in the group treated with GLB and in the group treated with BBE. The same results were observed by ethanolic extract of bee bread in our previous study (Bakour et al., 2017 ▶).

The results presented in Table 1 show that the bee bread is rich in antioxidants. Tocopherols and phenolic compounds profile of this bee bread was shown in a previous study which revealed a rich composition ofα-tocopherol, δ- tocopherol and flavonoids such as is orhamnetin, quercetin, kaempferol and methylherbacetin (Bakour et al., 2019 ▶).

It has been shown that ethyl acetate is a good solvent for extracting flavonoids (Amri and Hossain, 2018 ▶; Mujwah et al., 2010 ▶), therefore the biological effect of BBE could be attributed to its flavonoids content. Previous studies revealed that natural antioxidant compounds have beneficial effects on the treatment and prevention of diabetes and its complications; for instance, quercetin could reduce blood glucose levels in diabetic rats (Daniel et al., 2018 ▶; Hamilton et al., 2018 ▶; Srinivasan et al., 2018 ▶). In another study, it was shown that rutin has the ability to reduce liver injury induced by diabetes (Liang et al., 2018 ▶).

## References

[B1] Amri FSA, Hossain MA (2018). Comparison of total phenols, flavonoids and antioxidant potential of local and imported ripe bananas. Egypt J Basic Appl Sci.

[B2] Bakour M, Al-Waili NS, El Menyiy N, Imtara H, Figuira AC, Al-Waili T, Lyoussi B (2017). Antioxidant activity and protective effect of bee bread (honey and pollen) in aluminum-induced anemia, elevation of inflammatory makers and hepato-renal toxicity. J Food Sci Technol.

[B3] Bakour M, Fernandes Â, Barros L, Sokovic M, Ferreira ICFR, Lyoussi B (2019). Bee bread as a functional product: Chemical composition and bioactive properties. LWT.

[B4] Barene I, Daberte I, Siksna S (2014). Investigation of bee bread and development of its dosage forms. Med Teor Ir Prakt.

[B5] Chandramohan G, Al-Numair KS, Sridevi M, Pugalendi KV (2010). Antihyperlipidemic activity of 3-hydroxymethyl xylitol, a novel antidiabetic compound isolated from Casearia esculenta (Roxb ) root, in streptozotocin-diabetic rats. J Biochem Mol Toxicol.

[B6] Chatterjee S, Khunti K, Davies MJ (2017). Type 2 diabetes. The Lancet.

[B7] Chukwunonso Obi B, Chinwuba Okoye T, Okpashi VE, Nonye Igwe C, Olisah Alumanah E (2015). Comparative study of the antioxidant effects of metformin, glibenclamide, and repaglinide in alloxan-induced diabetic rats. J Diabetes Res.

[B8] Daniel OO, Adeoye AO, Ojowu J, Olorunsogo OO (2018). Inhibition of liver mitochondrial membrane permeability transition pore opening by quercetin and vitamin E in streptozotocin-induced diabetic rats. Biochem Biophys Res Commun.

[B9] de Wet H, Proks P (2015). Molecular action of sulphonylureas on KATP channels: a real partnership between drugs and nucleotides. Biochem Soc Trans.

[B10] DiMeglio LA, Evans-Molina C, Oram RA (2018). Type 1 diabetes. The Lancet.

[B11] Dimo T, Rakotonirina SV, Tan PV, Azay J, Dongo E, Kamtchouing P, Cros G (2007). Effect of Sclerocarya birrea (Anacardiaceae) stem bark methylene chloride/methanol extract on streptozotocin-diabetic rats. J Ethnopharmacol.

[B12] Dolezal AG, Toth AL (2018). Feedbacks between nutrition and disease in honey bee health. Curr Opin Insect Sci.

[B13] El Hilaly J, Lyoussi B (2002). Hypoglycaemic effect of the lyophilised aqueous extract of Ajuga iva in normal and streptozotocin diabetic rats. J Ethnopharmacol.

[B14] Erejuwa OO, Nwobodo NN, Akpan JL, Okorie UA, Ezeonu CT, Ezeokpo BC, Nwadike KI, Erhiano E, Abdul Wahab MS, Sulaiman SA (2016). Nigerian honey ameliorates hyperglycemia and dyslipidemia in alloxan-induced diabetic rats. Nutrients.

[B15] Farokhi F, Kaffash Farkhad N, Togmechi A, Soltani Band K (2012). Preventive effects of Prangos ferulacea (L ) Lindle on liver damage of diabetic rats induced by alloxan. Avicenna J Phytomedicine.

[B16] Garg A (1994). Management of dyslipidemia in IDDM patients. Diabetes Care.

[B17] Hamilton KE, Rekman JF, Gunnink LK, Busscher BM, Scott JL, Tidball AM, Stehouwer NR, Johnecheck GN, Looyenga BD, Louters LL (2018). Quercetin inhibits glucose transport by binding to an exofacial site on GLUT1. Biochimie.

[B18] Kazemian M, Abad M, Haeri MR, Ebrahimi M, Heidari R (2015). Anti-diabetic effect of Capparis spinosa L root extract in diabetic rats. Avicenna J Phytomedicine.

[B19] Kieliszek M, Piwowarek K, Kot AM, Błażejak S, Chlebowska-Śmigiel A, Wolska I (2018). Pollen and bee bread as new health-oriented products: A review. Trends Food Sci Technol.

[B20] Kong KW, Mat-Junit S, Aminudin N, Ismail A, Abdul-Aziz A (2012). Antioxidant activities and polyphenolics from the shoots of Barringtonia racemosa ( ) Spreng in a polar to apolar medium system. Food Chem.

[B21] Li Y, Wen S, Kota BP, Peng G, Li GQ, Yamahara J, Roufogalis BD (2005). Punica granatum flower extract, a potent α-glucosidase inhibitor, improves postprandial hyperglycemia in Zucker diabetic fatty rats. J Ethnopharmacol.

[B22] Liang W, Zhang D, Kang J, Meng X, Yang J, Yang L, Xue N, Gao Q, Han S, Gou X (2018). Protective effects of rutin on liver injury in type 2 diabetic db/db mice. Biomed Pharmacother.

[B23] Mărghitaş LA, Stanciu OG, Dezmirean DS, Bobiş O, Popescu O, Bogdanov S, Campos MG (2009). In vitro antioxidant capacity of honeybee-collected pollen of selected floral origin harvested from Romania. Food Chem.

[B24] Miguel M da G, Doughmi O, Aazza S, Antunes D, Lyoussi B (2014). Antioxidant, anti-inflammatory and acetylcholinesterase inhibitory activities of propolis from different regions of Morocco. Food Sci Biotechnol.

[B25] Mujwah AA, Mohammed MA, Ahmed MH (2010). First isolation of a flavonoid from Juniperus procera using ethyl acetate extract. Arab J Chem.

[B26] Nandhini ATA, Thirunavukkarasu V, Anuradha CV (2004). Stimulation of glucose utilization and inhibition of protein glycation and AGE products by taurine. Acta Physiol Scand.

[B27] Padmanabhan P, Jangle SN (2012). Evaluation of DPPH radical scavenging activity and reducing power of four selected medicinal plants and their combinations. Int J Pharm Sci Drug Res.

[B28] Pari L, Venkateswaran S (2004). Protective role of Phaseolus vulgaris on changes in the fatty acid composition in experimental diabetes. J Med Food.

[B29] Postic C, Dentin R, Girard J (2004). Role of the liver in the control of carbohydrate and lipid homeostasis. Diabetes Metab.

[B30] Ren B, Qin W, Wu F, Wang S, Pan C, Wang L, Zeng B, Ma S, Liang J (2016). Apigenin and naringenin regulate glucose and lipid metabolism, and ameliorate vascular dysfunction in type 2 diabetic rats. Eur J Pharmacol.

[B31] Silva TMS, dos Santos FP, Evangelista-Rodrigues A, da Silva EMS, da Silva GS, de Novais JS, dos Santos F de AR, Camara, CA (2013). Phenolic compounds, melissopalynological, physicochemical analysis and antioxidant activity of jandaíra (Melipona subnitida) honey. J Food Compos Anal.

[B32] Singh V, Singh SP, Singh M, Gupta AK, Kumar A (2015). Combined potentiating action of phytochemical(s) from Cinnamomum tamala and Aloe vera for their anti-diabetic and insulinomimetic effect using In Vivo rat and in vitro NIH/3T3 cell culture system. Appl Biochem Biotechnol.

[B33] Srinivasan P, Vijayakumar S, Kothandaraman S, Palani M (2018). Anti-diabetic activity of quercetin extracted from Phyllanthus emblica L fruit: In silico and in vivo approaches. J Pharm Anal.

[B34] Szkudelski T (2001). The mechanism of alloxan and streptozotocin action in B cells of the rat pancreas. Physiol Res.

[B35] Vaudo AD, Tooker JF, Grozinger CM, Patch HM (2015). Bee nutrition and floral resource restoration. Curr Opin Insect Sci.

[B36] Zarei A, Vaezi G, Malekirad AA, Abdollahi M (2015). Effects of ethanol extract of Salvia hydrangea on hepatic and renal functions of streptozotocin-induced diabetic rats. Avicenna J Phytomedicine.

[B37] Zengin G, Arkan T, Aktumsek A, Guler GO, Cakmak YS (2013). A study on antioxidant capacities and fatty acid compositions of two Daphne species from Turkey: New sources of antioxidants and essential fatty acids: antioxidant capacity and fatty acid composition. J Food Biochem.

